# Ocular biomarkers of cognitive decline based on deep-learning retinal vessel segmentation

**DOI:** 10.1186/s12877-023-04593-8

**Published:** 2024-01-06

**Authors:** Rui Li, Ying Hui, Xiaoyue Zhang, Shun Zhang, Bin Lv, Yuan Ni, Xiaoshuai Li, Xiaoliang Liang, Ling Yang, Han Lv, Zhiyu Yin, Hongyang Li, Yingping Yang, Guangfeng Liu, Jing Li, Guotong Xie, Shouling Wu, Zhenchang Wang

**Affiliations:** 1 Department of Radiology, Beijing Friendship Hospital, Capital Medical University, Beijing, China; 2 Ping An Healthcare Technology, Beijing, China; 3 Department of Psychiatry, Kailuan Mental Health Centre, Hebei province, Tangshan, China; 4 School of Public Health, North China University of Science and Technology, Hebei province, Tangshan, China; 5 Longzhen Senior Care, Beijing, China; 6 Department of Ophthalmology, Beijing Friendship Hospital, Capital Medical University, Beijing, China; 7 Ministry of Education Key Laboratory of Environment and Health, and State Key Laboratory of Environmental Health (Incubating), School of Public Health, Tongji Medical College, Huazhong University of Science and Technology, Wuhan, China; 8 Department of Ophthalmology, Peking University International Hospital, Beijing, China; 9 Department of Cardiology, Kailuan General Hospital, 57 Xinhua E Rd, Tangshan, China

**Keywords:** Retinal microvascular parameters, Cognitive function, Deep learning segmentation, Fundus photography

## Abstract

**Background:**

The current literature shows a strong relationship between retinal neuronal and vascular alterations in dementia. The purpose of the study was to use NFN+ deep learning models to analyze retinal vessel characteristics for cognitive impairment (CI) recognition.

**Methods:**

We included 908 participants from a community-based cohort followed for over 15 years (the prospective KaiLuan Study) who underwent brain magnetic resonance imaging (MRI) and fundus photography between 2021 and 2022. The cohort consisted of both cognitively healthy individuals (*N* = 417) and those with cognitive impairment (*N* = 491). We employed the NFN+ deep learning framework for retinal vessel segmentation and measurement. Associations between Retinal microvascular parameters (RMPs: central retinal arteriolar / venular equivalents, arteriole to venular ratio, fractal dimension) and CI were assessed by Pearson correlation. *P* < 0.05 was considered statistically significant. The correlation between the CI and RMPs were explored, then the correlation coefficients between CI and RMPs were analyzed. Random Forest nonlinear classification model was used to predict whether one having cognitive decline or not. The assessment criterion was the AUC value derived from the working characteristic curve.

**Results:**

The fractal dimension (FD) and global vein width were significantly correlated with the CI (*P* < 0.05). Age (0.193), BMI (0.154), global vein width (0.106), retinal vessel FD (0.099), and CRAE (0.098) were the variables in this model that were ranked in order of feature importance. The AUC values of the model were 0.799.

**Conclusions:**

Establishment of a predictive model based on the extraction of vascular features from fundus images has a high recognizability and predictive power for cognitive function and can be used as a screening method for CI.

**Supplementary Information:**

The online version contains supplementary material available at 10.1186/s12877-023-04593-8.

## Background

The aging population is growing all across the world. According to the United Nations, this trend will accelerate in the next decades, owing primarily to an increase in average life expectancy [1]. Cognitive decline significantly impacts activities of daily living, especially in the elderly. The incidence of cognitive decline ranged from 22 to 76.8 per 1000 person-years, with a median of 53.97 per 1000 person-years [2]. Various etiologies cause cognitive decline, including vascular diseases, neuronal degeneration, and stroke. Among these, alterations in cerebral microcirculation play a critical pathogenic role [3]. However, the existing limitations on MRI scan accessibility and the spatial resolution of neuroimaging methods prevent the clear visualization of the brain’s small vasculature.

The retinal microvasculature shares similar embryological origins and physiological characteristics with cerebral small vessel. Therefore, ocular examinations can be used as a noninvasive screening tool to investigate pathological changes in brain microcirculation and relevant cognitive impairment [4]. Retinal photography allows direct observation of retinal vessels and offers a straightforward, noninvasive method for assessing early changes in human brain circulation [5, 6]. As a result, it is widely accepted that pathophysiological processes affecting the central nervous system and cerebral microcirculation also profoundly influence the retina and its microcirculation.

With advanced retinal imaging technologies facilitating non-invasive assessment of the association between retinal microvascular parameters (RMPs) and cognitive function [7, 8]. Previous studies employing retinal photography have demonstrated a link between the presence of retinal microvascular abnormalities (e.g., fractal dimension, retinal vessel tortuosity) and cognitive dysfunction [9, 10]. However, in the Northern Ireland Cohort (an older community-based cohort), researchers failed to detect any associations between RMPs and those in the early stage of cognitive loss [11]. Moreover, although many studies have examined this relationship, only a limited number have prospectively investigated the link between retinal vessel abnormalities and the risk of cognitive dysfunction, and their findings have been inconsistent [12]. In a prospective study [10], researchers demonstrated that retinal vessel caliber measured by a deep learning algorithm on retinal photographs was associated with the risk of cognitive decline and incident dementia. Although, our study is a cross sectional study, if we could detect cognitive impairment people by using retinal imaging and retinal vessel assessment, this will support the idea convincingly that as a non-invasive potential tool, retinal vessel caliber may be invaluable to screen cognitive decline patient.

In a previous study, using deep learning-based vessel segmentation and measurement, FD, CRAE, CRVE and AVR were all found to decrease significantly with the progression of Diabetic Retinopathy (DR). Meanwhile, only FD significantly correlated with renal function indicators. Therefore, the FD should be regarded as a retinal vessel metric with great clinical significance [13]. Hence, in this study, we emphatically discussed if FD could be a valuable biomarker for cognitive impairment. Compared to traditional machine learning methods, The NFN+ deep learning is an end-to-end framework which integrates feature extraction into learning, and has been proved to be the most suitable for large sample data [14]. Therefore, the aim of this study was to evaluate associations between retinal vessels characteristics and cognitive impairment in a community-based cohort and to explore a convenient method to identifing individuals at high risk for cognitive decline.

## Methods

### Participants

**Fig. 1:**
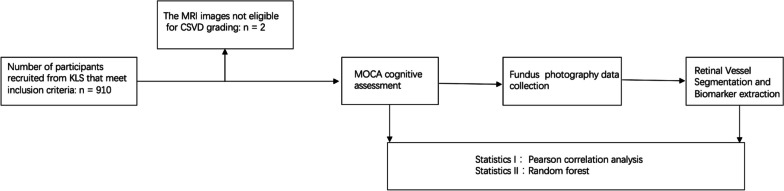
Flow chart of patient inclusion

Nine hundred ten subjects were recruited from the Kailuan community in Tangshan, Northern China, between January 2020 and August 2022. Out of these 910 participants, 2 had MRI examination that the images not eligible for cerebral small-vessel disease (CSVD) grading. The CSVD is composed of several diseases affecting the small arteries, arterioles, venules, and capillaries of the brain, and refers to several pathological processes and etiologies. This resulted in a final sample size of 908 participants (Fig. 1). The study was approved by the Medical Ethics Committee of Kailuan General Hospital. The research followed the principles of the Helsinki Declaration, and all subjects provided written informed permission. Participants were eligible if they were aged between 23 and 83 years (all gender), without contra-indications of retinal fundus photograph, had adequate language abilities for a neuropsychological evaluation, and could cooperate with clinical data collection. The exclusion criteria were congenital or acquired organic illnesses history, a clinical history of mental illnesses, cognitive impairment due to epilepsy, multiple sclerosis, traumatic brain injury, refractive error, or substance abuse disorders. The enrolled subjects’ ophthalmic characteristics are described in Supplementary Table 1.

### Measurement of cognitive function

Cognitive function was evaluated using the Montreal Cognitive Assessment (MoCA), a widely-used screening test for identifying cognitive dysfunction [15].

The one-page, 30-point assessment can be completed in around 10 minutes. The MoCA total score ranges from 0 to 30, with lower scores (<26 points) indicating worse cognitive capacity. The test was conducted by an expert examiner.

### Measurement of retinal images

The bilateral fundus photography images were obtained by a fundus camera (CX-1, Topcon, Topcon Corporation, Tokyo, Japan).

Quality control criteria were as follows: (1) no severe artifact or blurring; (2) brightness that was not too dark or too light; and (3) the image field contained the entire optic disc and macula.

Low-quality images were discarded. If an image was ungradable or could not be measured accurately, the other eye was used for evaluation. If both eyes have poor quality images, then that patient was excluded from this study.

### Retinal vessel segmentation via deep learning

**Fig. 2:**
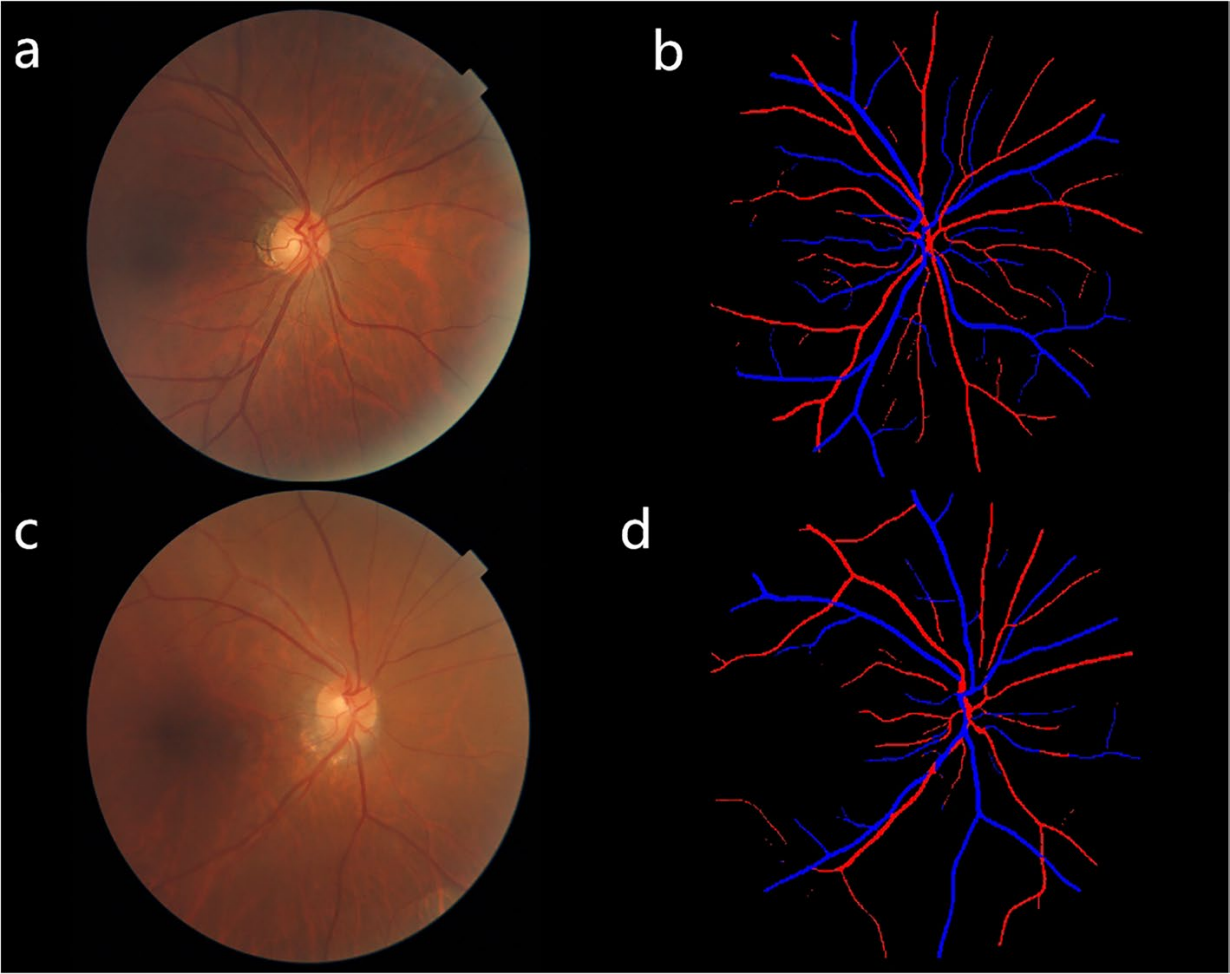
Examples of retinal vessel segmentation results, a and b are images without cognitive impairment, c and d are images with cognitive impairment. Examples of retinal vessel segmentation results. **a**, **c** Original fundus image. **b**, **d** Segmented arteries and veins: arteries shown in red, and veins in blue

We realized the automatic extraction of retinal vesselusing a recently published deep learning based approach called NFN+. The proposed NFN+ model is novel in the cascaded design and inter-network skip connections. Two cascaded identical backbones, i.e., a front network and a followed network, are united by inter-network skip connections. The front network takes image patches as its input and produces prime vessel probability maps. The followed network takes the prime vessel probability maps produced by the front network as its input and generates vessel segmentation results [16] (Fig. 2). Based on NFN+ model, we use vignetting mask strategy to improve the performance of vessel segmentation for our collected images in clinical use scenarios [14]. The doctor manually marks the arteries and veins according to the segmentation results. Then we measured a vessel geometric class called retinal vessel FD. The FD is a global measure to quantify the physiologic complexity, including the whole vascular networks. Using the box-counting method, each segmented mask was divided into a series of squares of various side lengths along the centerline tracings of retinal vessels. The FD was then defined as the gradient of logarithms of the number of boxes and the size of those boxes. The more complex the branching pattern, the greater the FD. Besides the vessel geometric class, we also measured the diameters of all arterioles and venules coursing through an area of 0.50-to-1-disc diameter surrounding the optic disc. The Knudtson– Hubbard formula was used to calculate average retinal arteriolar and venular caliber, named as CRAE and CRVE, respectively. And the AVR is the ratio of CRAE to CRVE. Morphology thinning operation was performed on the arteries (veins) to obtain a skeleton map, then global artery (vein) width was got by whole artery (vein) area divided by artery (vein) skeleton length.

### Statistical analysis

All statistical analyses were conducted using SPSS statistics software version 22.0 and the R statistical package (version 3.6; The R Foundation, Vienna, Austria). Variables were analyzed by Mann–Whitney U, Student’s t-tests or chi-squared test, as appropriate. To further determine the relationships of clinical variables, we calculated correlation coefficients between the cognitive score (MoCA) and physiological indexes (including gender, age, BMI, history of hypertension, history of diabetes, history of hyperlipidemia), and vascular biomarker (including CRAE, CRVE, AVR, fractal dimension, global vein width, global artery width). A *P* value less than 0.05 was considered statistically significant. Linear regression was also used to investigate the correlation coefficients when they reached the statistical significance. The receiver operating characteristic (ROC) curve of FD and global vein width were drawn, and the optimal cut-off point of RMPs in participants with cognitive decline was predicted according to the maximum value of the Youden Index.

Random Forest nonlinear classification model was utilized to predict cognitive impairment. This algorithm belongs to the so-called ensemble-learning and to choose the prediction with the highest votes as the classification result. In this study, our model inputs were physiological indexes (gender, age, BMI, history of hypertension, history of diabetes, history of hyperlipidemia) and vascular biomarker (CRAE, CRVE, AVR, fractal dimension, global vein width, global artery width). To further investigate the importance of the vascular biomarkers, we compared our model with the model considering physiological indexes alone. The model output was whether having mild cognitive impairment. The evaluation standard was the AUC value derived from the working characteristic curve.

## Results

Table 1 shows the baseline characteristics of participants with and without significant cognitive decline. There were no differences in smoking status, alcohol consumption, diabetes, mean BMI or prevalence of hyperlipidemia between the groups. However, subjects with cognitive decline were, on average, older, less educated, and had a higher CSVD score than those without.

**Table 1:** Participant summary characteristics

Patient characteristics	All (*n* = 908)	With cognitive decline (*n* = 491)	Without cognitive decline (*n* = 417)	*P* value	z value
Mean age (years, SD)	55.53	59.68 ± 10.730	50.65 ± 11.397	< 0.001	–
Female, n(%)	493	295 (60.1%)	198 (47.5%)	< 0.001	–
Smoking status, yes n (%)	221 (24.4%)	130 (26.5%)	91 (21.8%)	0.104	–
Alcohol consumption, non-drinker, n (%)	679 (74.9%)	362 (73.7%)	317 (76.0%)	0.490	–
Education	4.00 (3.00, 5.00)	4.00 (3.00, 5.00)	5.00 (4.00, 5.00)	< 0.001	−11.302
Diabetes, yes n (%)	100 (11%)	58 (11.8%)	42 (10%)	0.457	–
Mean BMI (kg/m2, SD)	25.21 ± 3.40	25.37 ± 3.25	25.01 ± 3.56	0.113	–
Cardiovascular disease, n (%)	2 (0.2%)	2 (0.4%)	0	0.503	–
Hypertension n (%)	433 (47.7%)	265 (54.0%)	168 (40.3%)	< 0.001	–
Hyperlipidemia, n (%)	125 (13.8%)	76 (15.5%)	49 (11.8%)	0.102	–
Mean MOCA test score (SD)	24.49 ± 3.84	21.78 ± 3.148	27.66 ± 1.318	< 0.001	–
CSVD	–	–	–	< 0.001	94.723
0	296 (32.6%)	101 (20.6%)	195 (46.8%)	–	–
1	271 (29.8%)	146 (29.7%)	125 (30.0%)	–	–
2	190 (20.9%)	126 (25.7%)	64 (15.3%)	–	–
3	90 (9.9%)	65 (13.2%)	25 (6.0%)	–	–
4	61 (6.7%)	52 (10.6%)	9 (2.2%)	–	–
Medication use					
Hypoglycemic agents, n (%)	59 (6.5%)	26 (5.3%)	33 (7.9%)	0.137	
Antihypertensive medication, n (%)	316 (34.8%)	166 (33.8%)	150 (36.0%)	0.529	
Lipid-modifying medication, n (%)	66 (7.3%)	35 (7.1%)	31 (7.4%)	0.898	
AVR	0.722 ± 0.128	0.714 ± 0.142	0.732 ± 0.108	0.030	
CRAE	155.048 ± 28.619	151.748 ± 29.719	158.862 ± 26.864	<0.001	
CRVE	217.068 ± 34.726	214.560 ± 37.510	219.977 ± 31.034	0.019	
FD	1.305 ± 0.084	1.289 ± 0.090	1.324 ± 0.074	<0.001	
Global artery width	79.248 ± 12.853	78.711 ± 13.701	79.777 ± 11.633	0.205	
Global vein width	127.703 ± 28.889	133.062 ± 33.319	121.497 ± 21.074	<0.001	

Among the correlation analysis between cognitive states and retinal vessel metrics, the FD positively correlated with cognitive decline (*r* = 0.26, *P* < 0.001). The optimal cut-off point of FD of the participants with cognitive decline was 1.336, and the area under the curve (AUC) was 0.636 (sensitivity, 60.7%; specificity, 61.7%). Additionally, global vein width (*r* = − 0.2, *P* < 0.001) also showed a significant correlation with cognitive impairment (Table 2). The global vein width of subjects with cognitive impairment were optimally cut-off at 122.81, with an area under the curve (AUC) of 0.615 (sensitivity, 63.6%; specificity, 55.6%).

**Table 2:** Correlations of retinal vascular metrics Clinical, Characteristics and cognitive impairment

Feature	Pearson value	*p* value
avr	0.08	< 0.001
crae	0.16	< 0.001
crve	0.11	< 0.001
df	0.26	< 0.001
age	−0.47	< 0.001
female	0.12	< 0.001
Global artery width	0.01	0.6056
Global vein width	−0.21	< 0.001
hypertension	−0.17	< 0.001
diabetes	−0.12	< 0.001
hyperlipidemia	−0.06	0.0012

**Fig. 3:**
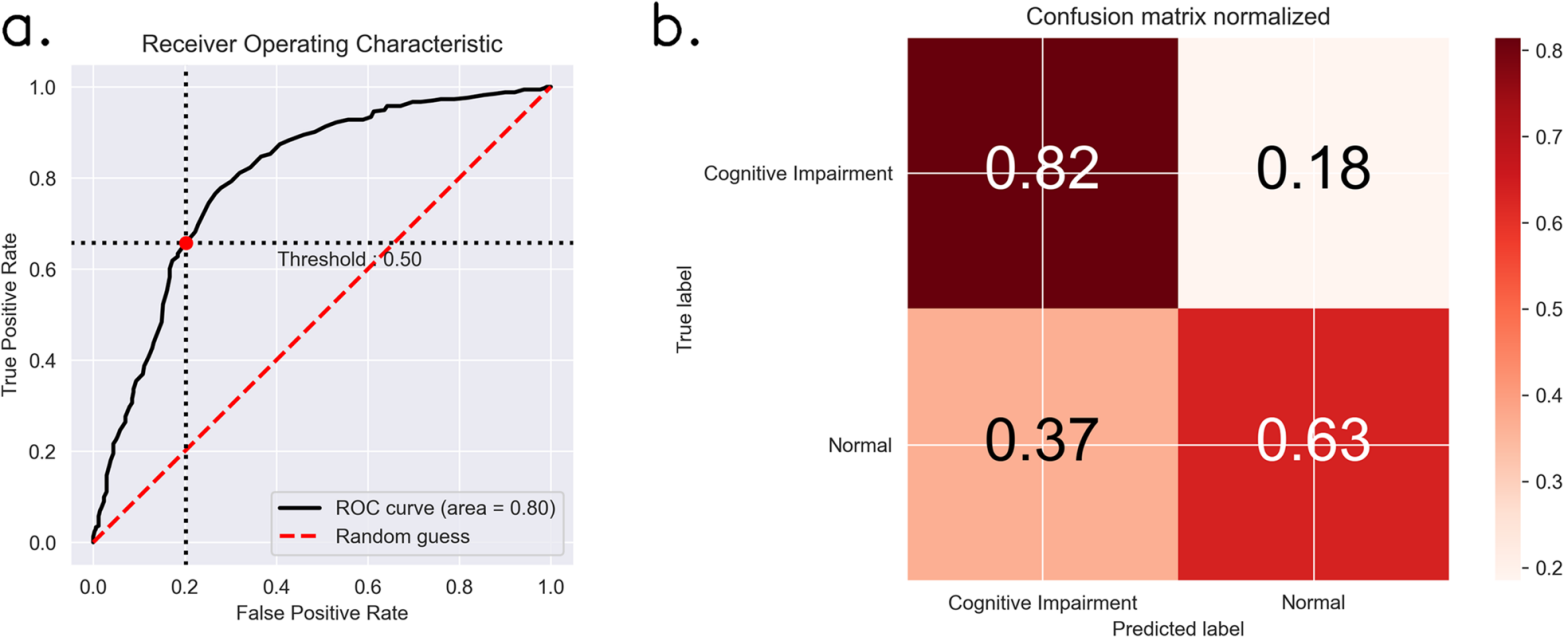
Model performance on test set. **a** shows the receiver operating characteristic curves with black line representing our model and red dashed line representing the random guess. and **b** shows the normalized confusion matrices of our model

**Fig. 4:**
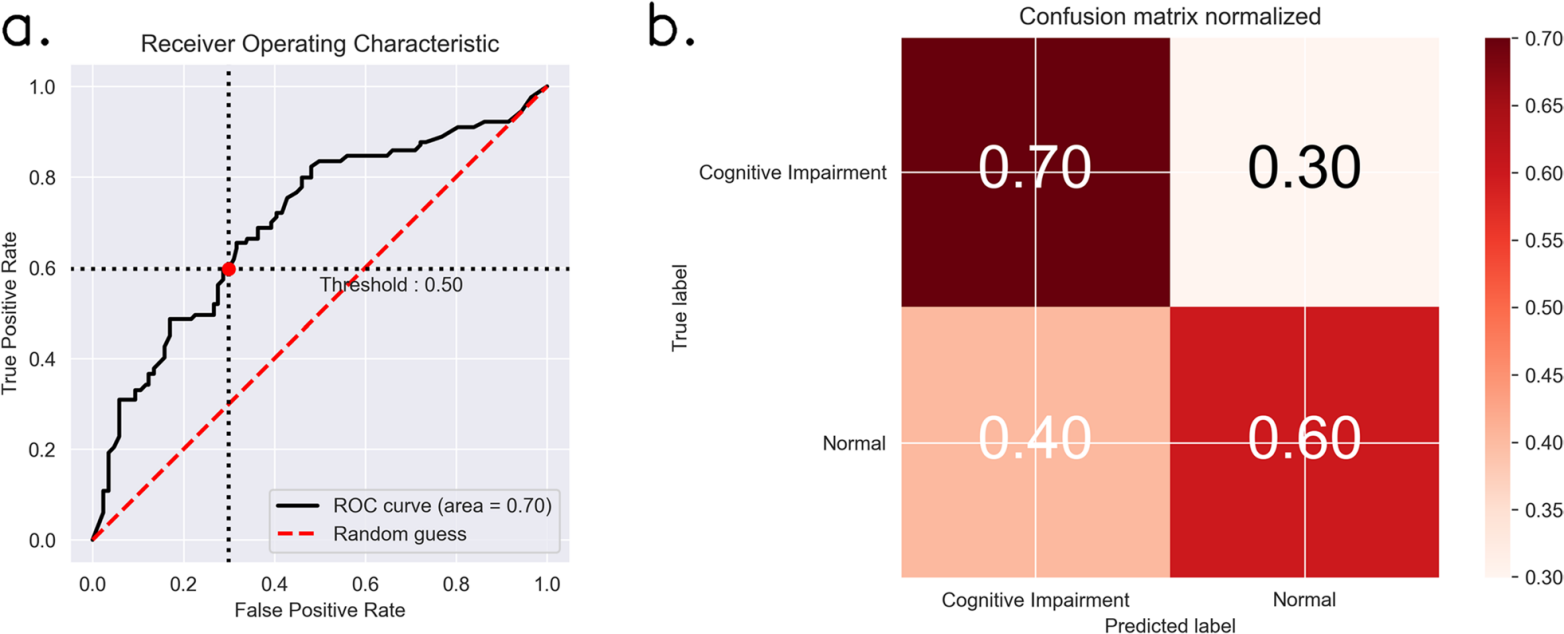
Performance of the model with physiological indexes alone on test set. **a** shows the receiver operating characteristic curves with black line representing our model and red dashed line representing the random guess. And (**b**) shows the normalized confusion matrices of our model

The patients were divided into the training set and the test set according to the ratio of nearly 4:1. The participants’ demographic and clinical characteristics for both the training and test sets were showed in supplement Table 2. In the training set, the ratio of cognitive impairment to normal was about 1:1. The order of feature importance of predictors in this model was age (0.193), BMI (0.154), global vein width (0.106), retinal vessel FD (0.099), and CRAE (0.098), the AUC values predicted by the model were 0.799 (Fig. 3). For the model with physiological indexes alone, the AUC was 0.70 (Fig. 4).

## Discussion

In the current research, retinal biomarkers characterizing the structure of the retina were compared to evaluate retinal vessel alterations in participants with different cognitive states. Our results show a trend of a positive correlation between cognitive function and retinal vessel FD. In contrast, the correlation between cognitive function and global vein width is negative. The study used random forest to predict the development of cognitive decline. The order of importance of predictors in the model was as follows: age (0.193), BMI (0.154), global vein width (0.106), retinal vessel FD (0.099), and CRAE (0.098). The AUC values predicted by the model were 0.799.

A key feature of our study is that the Kailuan community included participants of a wide age range. This allowed for a more accurate age-stratified analysis, making it easier to recognize cognitive decline. Cognitive impairment is a serious medical, social, and economic public health problems that have a considerable impact on older individuals’ quality of life [17]. Several clinical tools are available for screening cognitive impairment. The most widely used tool for screening cognitive impairment is the Mini-mental State Examination (MMSE). However, it has proven inadequate for detecting mild cognitive impairment (MCI) and clinical indications of dementia because of its ceiling effect [18]. Our study used the MoCA to evaluate cognitive function. The MoCA was primarily designed as a valid and reliable MCI screening tool, with higher sensitivity and specificity than the MMSE [19]. Previous literature reports that the MoCA’s sensitivity for identifing MCI is 90% [20]. However, MoCA may not provide a complete evaluation of cognitive function. Additionally, various neuroimaging techniques, such as arterial spin labelling (ASL), diffusion tensor imaging (DTI), magnetic resonance spectroscopy (MRS),are used in clinical practice. Many studies have reported the use of MRI as a biomarker for diagnosing MCI or dementia [21, 22]. However, MRI facility is difficult to effectively carry out largescale screening cognitive impairment, and it does not offer microstructural details on the cerebral microvasculature.

Pathological alterations in the cerebral microcirculation play a critical pathogenic role in cognitive impairment [23]. The retina, part of the central nervous system (CNS), shares many similarities with the brain in its developmental history, anatomical structure, and physiological characteristics [24]. Therefore, retinal vessels provide a special and convenient “window” for studying brain microvascular disease, enabling non-invasive in vivo observation of human microcirculation. Retinal examination is simple to use, economical, and many hospitals have progressively incorporated it into physical screening programs or made it more widely known through social health initiatives [25]. Retinal photography allows for direct observation of retinal vessels, and some quantitative parameters reflecting retinal blood vessel changes can be obtained through software package. With the booming development of deep learning, many recent algorithms exhibited excellent performance on retinal vessels. Based on these segmentation results, correlations between blood vessel morphology and diseases are able to be analyzed. Previously, our team developed the NFN+ method, combined with the vignetting mask strategy to achieve retinal vessel segmentation and evaluated it on randomly 50 fundus images from the collected dataset [14, 16]. Leveraging this algorithm, we found relationships between retinal vessel characteristics and renal function in patients with type 2 diabetes mellitus. In this study, more characteriscts are included based on deep learning segmentation to analysis the relationship between retinal vessel and cognitive decline.

The global vein width represents the evaluation of the whole retinal vein using deep learning techniques. To our knowledge, there are few reports on the relationship between global vein width and cognitive function. Most studies have focused on the central retinal venular equivalents and cognitive function [11, 12]. However, recent research findings suggest that small vessels carry more information for diagnosing Alzheimer’s diseases (AD) [26]. Therefore, we obtained the mean width of small arteries and veins by software analysis, and the study indicated that a significant negative correlation between global vein width and cognitive function. The wider retinal venular caliber may be associated with cognitive decline. Prior evidence indicates that [27] the wider retinal venular calibers are more related to cerebral hypoperfusion and cerebrovascular disease, both of which can cause cognitive decline. Nevertheless, these findings require replication in other studies. Studies have reported a strong association between narrower retinal arteriolar calibers and conditions such as hypertension and dementia [12, 27]. However, the results of this study showed that CRAE and global arterial width did not significantly correlate with cognitive function. The possible reason for this discrepancy might be the different cognitive assessment tools and outcome definitions used across studies, make it difficult to directly compare results.

Fractal dimension [28] is used to measure the complexity of the vascular tree and quantitatively summarizes the global structure of the retinal circulation. The primary benefit of FD is that it is unaffected by image magnification caused by retinography or eye refractive characteristics. It is a “global” metric that takes into account every branch of the retinal vessel tree within a single parameter [29]. A reduced vascular FD in the retina shows vessel rarefaction and collapse caused by hypoxia. Less branching density, which is known to develop in various organ regulatory systems of the aging human body, is represented by a reduced fractal dimension. Based on a number of clinical trials, a reduced retinal vessel fractal dimension has been linked to hypertension [30], stroke [31], and AD [32]. In our study, decreased retinal vessel fractal dimension is associated with cognitive dysfunction, which is consistent with the current literature [29, 33, 34]. However, O’Neill et al. [11] discovered that no significant associations between FD and cognitive dysfunction. A possible reason for this discrepancy might be that our study is focused on Asian population with a wide age range, while the NICOLA study focused on the Northern Ireland community population aged 50 years and older. Additionally, significant intersoftware differences in retinal vessel parameters have been found in several studies [23], which can also cause differences in results. Furthermore, our research did not distinguish arterial and venular FD, Williams et al. [32] found that lower venular FD in Alzheimer’s disease, in contrast, the association between arteriolar FD and Alzheimer disease was not reported in their models. Therefore, we believe that the venular FD will be more sensitive and the future work is needed to test.

There is a great deal of interest in finding sensitive biomarkers that shed light on the structural and functional pathophysiological changes affecting the brain. Early diagnosis is essential for initiating timely and optimal management of cognitive impairment and for preventing or delaying disease progression [35, 36]. Currently, Random Forest [37] is widely used in the development of medical prediction models with a large number of predictor datasets, its excellent data processing capability and predictive performance are increasingly recognized by scholars. In this study, random forest was applied to predict the risk factors of cognitive decline in Kailuan popoulation. Five significant predictors were found, including: age, BMI, global venous width, FD, CRAE. As previous studies stated [38], a positive association between age and cognitive dysfunction even adjusting for other characteristics in a multivariable logistic regression model. Therefore, it is not surprising that age is a significant predictor of cognitive decline. A study of BMI and cognitive function in a Chinese population found that [39] a reverse J-shaped association was observed between BMI and cognitive impairment. Our study further confirms that BMI is a significant predictor of cognitive decline. Researches [23, 24, 34] have reported an association between retinal microvascular parameters and cognitive dysfunction. Therefore, we incorporated RMPs and clinical feature parameters into a random forest prediction model. The findings indicated that, compared to models employing only clinical feature parameters, the combined model’s prediction accuracy was enhanced.

There are several limitations to our study that may represent a direction for further research. First, our investigation lacks longitudinal data, which would ascertain the accuracy of retinal biomarkers in routinely identifying patients, even in preclinical stages before the manifestation of cognitive symptoms. Second, not only the retinal microvascular changes but also the abnormal bioelectrical activity of retinal ganglion cells, photoreceptors and the optic nerve have been associated with cognitive decline [40]. Additional multimodal mapping of retinal neurovascular measures will be conducted in our future research. Third, although retinal biomarkers are quite promising for cognitive impairment screening, there is a lack of data on their sensitivity and specificity. Furthermore, because this study was observational in nature, we are unable to extrapolate any conclusions about potential causal links between retinal vessel calibre and cognitive deterioration from these results.

## Conclusion

The FD and global vein width significantly associated with the CI. We used random forest nonlinear classification model to predict CI, age, BMI, global vein width, retinal vessel FD, and CRAE were the variables in this model that were ranked in order of feature importance. The AUC values of the model were 0.799. The NFN+ model, which can extract vascular features from fundus images, has a high level of recognition and predictive capacity for cognitive function and can be utilized as a screening approach for CI.

### Supplementary Information

**Additional file 1.**

## Data Availability

The datasets used and/or analyzed during the current study are available from the corresponding author on reasonable request.

## Abbreviations

CI: Cognitive impairment
MRI: Magnetic resonance imaging
RMPS: Retinal microvascular parameters
FD: Fractal dimension
CRVE: Central retinal venular equivalents
CRAE: Central retinal arteriolar equivalents
AVR: Arteriole to venular ratio
DR: Diabetic retinopathy
CSVD: Cerebral small-vessel disease
MoCA: Montreal Cognitive Assessment
MMSE: Mini-mental State Examination
MCI: Mild cognitive impairment
ASL: Arterial spin labelling
DTI: Diffusion tensor imaging
MRS: Magnetic resonance spectroscopy
CNS: Central nervous system
AD: Alzheimer’s diseases
