# Supplementary material for: Ocular biomarkers of cognitive decline based on deep-learning retinal vessel segmentation
Source: BMC Geriatr. 2024 Jan 6;24:28. doi: 10.1186/s12877-023-04593-8 (PMC10770952; doi:10.1186/s12877-023-04593-8)
Supplement: Supplementary file 1 — Additional file 1. [file 12877_2023_4593_MOESM1_ESM.docx]

Supplementary table1 participant ophthalmic characteristics

| Abnormal | Number |
| --- | --- |
| Drusen | 75 |
| Macular degeneration | 50 |
| EpiretinaI membrane | 45 |
| High Myopia | 13 |
| Macular vitreous warts | 11 |
| Microvesicle | 10 |
| Macular edema | 5 |
| Exudative | 5 |
| Vitreous Degeneration | 4 |
| Fundus Hemorrhage | 4 |
| Arteriovenous abnormal | 4 |
| Posterior vitreous detachment | 3 |
| Macular microvesicles | 3 |
| Vesicles | 3 |
| Irregular pigment epithelium | 3 |
| Cotton-wool spot | 2 |
| Branch retinaI vein occIusion | 2 |
| Fundus changes | 2 |
| Choroidal vascular atrophies | 2 |
| Cataracts | 1 |
| Large optic cup | 1 |
| Macular fissure | 1 |
| Macular pucker | 1 |
| Supratemporal retinal atrophy | 1 |
| Indistinct borders of the optic papilla | 1 |
| Retinopathy | 1 |
| Central retinal artery occlusion | 1 |
| Retinoschisis | 1 |
| Tilt of disk | 1 |
| Diabetic retinopathy | 1 |
| Ellipsoid zone absence | 1 |
| Microaneurysm，Exudative | 1 |
| Inner lamellar macular holes | 1 |
| Foveoschisis | 1 |

Supplementary table2 Training and test sets demographic and clinical characteristics

| Patient characteristics | All（n=908） | training set（n=727) | test set(n=181) | P value | z value |
| --- | --- | --- | --- | --- | --- |
| Mean age（years, SD） | 55.46±11.92 | 55.62±11.91 | 54.83±11.99 | 0.494 | - |
| Female, n(%) | 416（45.8%） | 330（45.4%) | 86(47.5%) | 0.618 | - |
| Education | 5.00 (3.00, 5.00) | 5.00 (5.00, 5.00) | 5.00 (4.00, 5.00) | 0.001 | -3.359 |
| Diabetes, yes n (%) | 100（11.0%） | 89（12.2%） | 11（6.1%） | 0.017 | - |
| Hypertension n (%) | 433（47.7%） | 345（47.5%） | 88（48.6%） | 0.803 | - |
| Hyperlipidemia, n (%) | 125（13.8%） | 102（14.0%） | 23（12.7%） | 0.718 | - |
| Mean MOCA test score (SD) | 25（22,27） | 25（22,27） | 25（23,28） | 0.094 | - |
